# COVID-19 in the Netherlands: lessons from a nationwide query of dutch autopsy, histology, and cytology pathological reports

**DOI:** 10.1007/s00428-024-03771-2

**Published:** 2024-02-27

**Authors:** Boaz Lopuhaä, Q. J. M. Voorham, Folkert J. van Kemenade, Jan H. von der Thüsen

**Affiliations:** 1 Department of Pathology and Clinical Bioinformatics, Erasmus University Medical Center, Doctor Molewaterplein 40, 3015 GD Rotterdam, the Netherlands; 2 Dutch Nationwide Pathology Databank (Palga), Houten, the Netherlands

**Keywords:** COVID-19, Autopsy, Thrombosis, Long COVID, Epidemiology

## Abstract

Since the onset of the COVID-19 pandemic, autopsies have played a valuable role in understanding the pathophysiology of COVID-19. In this study, we have analyzed COVID-19-related pathology reports from autopsies, histology, and cytology on a nationwide level. Pathology reports from all 43 pathology laboratories in the Netherlands stating “COVID,” “Corona,” and/or “SARS” were queried from the Dutch Nationwide Pathology Database (Palga). Consecutive reports of the included patients were also retrieved. Out of 5065 entries, a total of 1833 eligible COVID-19-related pathology reports between January 2020 and June 2021 were included in this collection of reports. Lung histopathology reports reflected differences in the severity of abnormalities (acute diffuse alveolar damage, alveolar histiocytes, and thrombi during the first three pandemic waves (Wuhan variant) versus the fourth wave (alpha variant)). Autopsy reports from 2020 state significantly shorter disease duration and younger age of death compared to autopsy reports from 2021. All reports together reflected a more granular pathology with comorbidities such as chronic histiocytic intervillositis, perniosis, and thrombi found in a variety of organs (lungs, kidneys, and small and large intestines). This nationwide overview of pathology reports provides data related to deaths as well as comorbidities in a clinical setting of COVID-19. Certain findings reported in SARS-CoV-infected lungs and placentas were also reported in post-COVID-19 tissue of the same kind. Consecutive reports after the earliest reports with COVID-19 allowed for follow-up reports. These follow-up reports can help with post-viral studies regarding long-term effects of COVID-19 as well as identifying the effects of different SARS-CoV-2 variants.

## Introduction

The recent COVID-19 pandemic has led to a substantial incidence of infections and subsequent deaths. By May 2023, over 765 million COVID-19 cases had been reported with a cumulative number of deaths passing 6.9 million [1]. Autopsies on COVID-19 patients have provided pathologists and clinicians with valuable information on the effects of acute, severe COVID-19 in lung tissue such as the presence of diffuse alveolar damage (DAD), pulmonary (micro)thrombi, and interstitial fibrosis [2–5]. Apart from acute effects, chronic effects of COVID-19 are already emerging. Reportedly, 2.3 to 36.5% of COVID-19 patients have long-lasting symptoms known as “long COVID” for at least 3 months [6–8]. Not only lungs are affected by COVID-19 as non-pulmonary comorbidities have been reported, such as gastrointestinal, dermal, and renal manifestations [9–11]. Although autopsy reports have provided researchers with critical information regarding acute COVID-19 pathophysiology, chronic effects of COVID-19 on lung tissue and other organs have not been investigated to the same extent. To gain insight in both acute and chronic effects of COVID-19 in lung tissue and other organs, we decided to analyze all pathology reports in the Netherlands of COVID-19 patients from autopsies, as well as histopathology and cytology requisitions during the clinical phase, including reports from former COVID-19 patients.

In the Netherlands, all pathology laboratories and hospitals require pathologists to code their reports so they can be stored in a national digital database, the Dutch Nationwide Pathology Database (Palga) [12]. On a national scale, all anonymized pathological reports with corresponding inquiries can be retrieved through Palga. This study reports on findings retrieved from pathology reports related to COVID-19 in the Netherlands, from autopsies as well as histology and cytology. The derived query result of reports has multiple purposes. Firstly, pathology reports provide data on COVID-19 pulmonary pathology together with unexpected manifestations in other organs. Secondly, this collection of reports provides epidemiological data regarding different variants of SARS-CoV-2 with corresponding pandemic waves, which may give insight in the prognosis of these variants. Lastly, consecutive reports of COVID-19 patients can be automatically identified by Palga. Data regarding chronic effects of COVID-19 and differences in severity and effects between known and potential new variants may accumulate over time. Thus, data from this query result serve as a basis for an ever-expanding database on COVID-19 pathophysiology and could possibly help in understanding long COVID.

## Methods

COVID-19-related excerpts from pathology reports between January 2020 and June 2021 in the Netherlands were retrieved from the Dutch Nationwide Pathology Database (Palga). Excerpts of reports contained the following information: macroscopic and microscopic description, patient age and sex, date of procedure, and report category (autopsy, histopathology, or cytology). The text in the initial request from the clinician was included if permission was granted by the laboratory/hospital where the pathology report originated from. Permission was granted by Palga review board, and the need for consent was waived since data are pseudonymized.

**Fig. 1:**
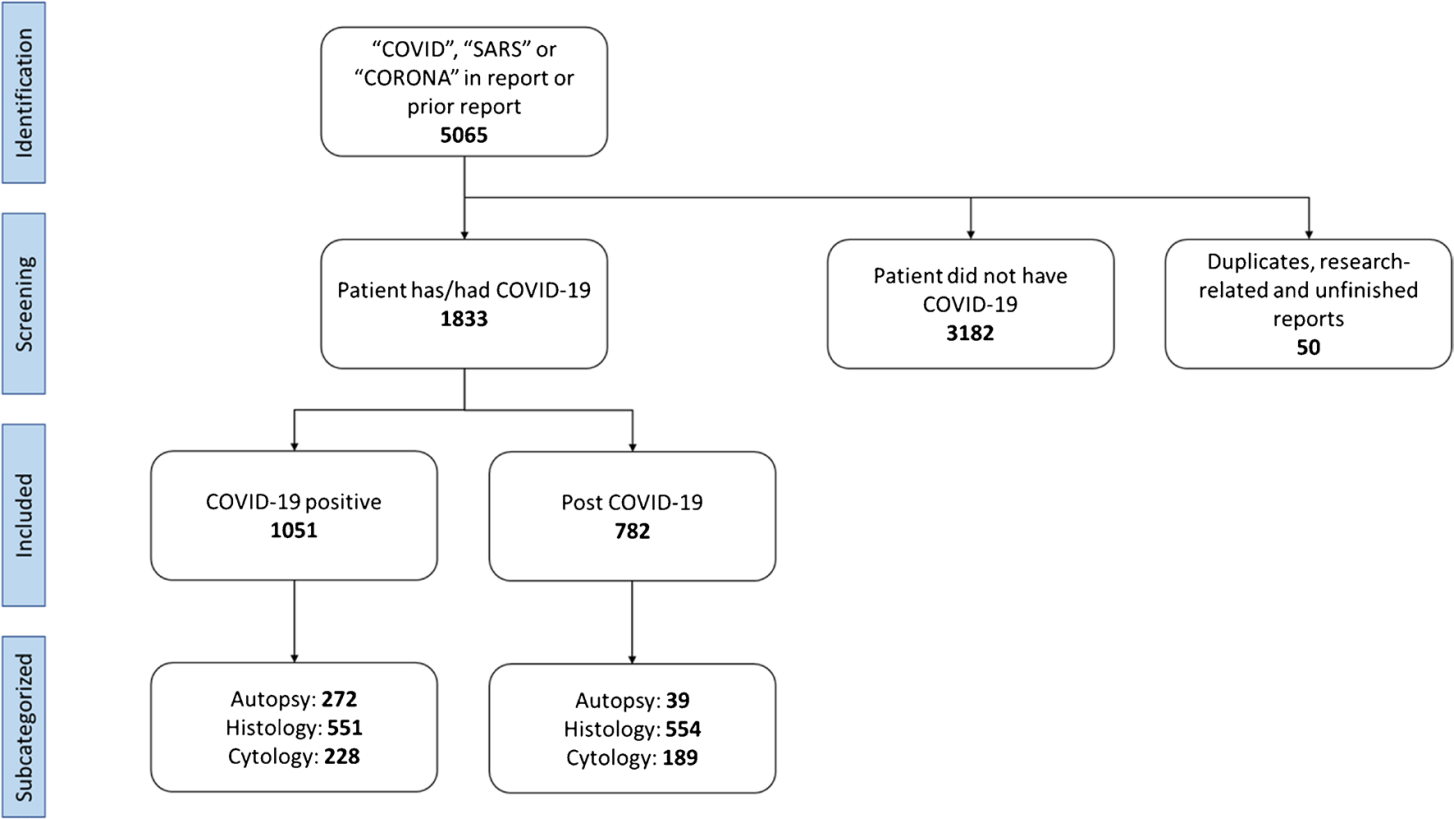
Flowchart of pathology report selection. Between January 2020 and June 2021, a total of 5065 reports were automatically selected if one of the following two criteria was met: either the report mentioned “Corona,” “COVID,” or “SARS”, or the report originated from patients included because of the first criterion. Reports were screened manually if the report or inquiry stated the patient had COVID-19. Reports without confirmation of COVID-19 were excluded (*n* = 3182) as well as duplicate reports, unfinished reports, and supplemental reports generated for research purposes (50). Included reports were divided into two groups; either reports stated that the concerned patient was COVID-19 positive at the time of the intervention (*n* = 1051) or had COVID-19 at least 1 month before the intervention (*n* = 782)

Pathology reports between January 2020 and June 2021 were retrieved from Palga if at least one of the following terms was mentioned in the report: “SARS,” “COVID,” or”CORONA”; or if the report was coded to indicate involvement of SARS-CoV-2. These reports were tagged by Palga so that subsequent reports from the same patients could be identified and prospectively included. Duplicate reports were removed. Next, reports and inquiries were manually selected and divided into two categories. The first category consisted of reports in which either the request or the report itself stated the patient had COVID-19 at the time of material retrieval (Fig. 1). The second category consisted of reports which stated patients either recovered from COVID-19 were discharged from a previous admission due to COVID-19, or if prior reports from the same patient ID stated they had COVID-19.

### Report information

Reports were categorized based on examination types (histopathology, cytology, and autopsy) and sample location. Autopsy reports were utilized to establish the duration of disease as determined by the interval between either the first date of COVID-19-related symptoms, positive COVID-19 test or admission (prioritized in this order), and death. Histomorphological characteristic findings for COVID-19 in lung tissue were recorded, such as DAD phase, (micro)thrombi, and pulmonary fibrosis. Cause of death was divided into two groups based on the inquiry and autopsy report: immediate and underlying cause of death. Immediate cause of death was the disease, injury, or complication directly causing death stated in the conclusion. Underlying cause of death, disease or injury that initiated the chain of events leading to death, was deduced from inquiry and conclusion.

## Results

Between January 2020 and June 2021, 5065 reports were archived which mentioned SARS, COVID, and/or CORONA (Fig. 1). These reports were derived from 43 laboratories or hospitals in the Netherlands and included reports from 3201 individual patients. Duplicate reports, incomplete reports, and non-diagnostic reports for research purposes were excluded (*n* = 50). Of the remaining 5015 reports, 1051 reports (21%) mentioned the corresponding patient was COVID-19 positive at the time of specimen acquisition, and 782 reports (16%) were taken at least 1 month after COVID-19 infection. The remaining 3182 reports were considered false positives and were excluded from this collection. For example, if the request mentioned “SARS-CoV-2 status: negative” in the request or if the conclusion stated “No indication for COVID-19 related pathology,” the report was excluded.

### Autopsies

**Fig. 2:**
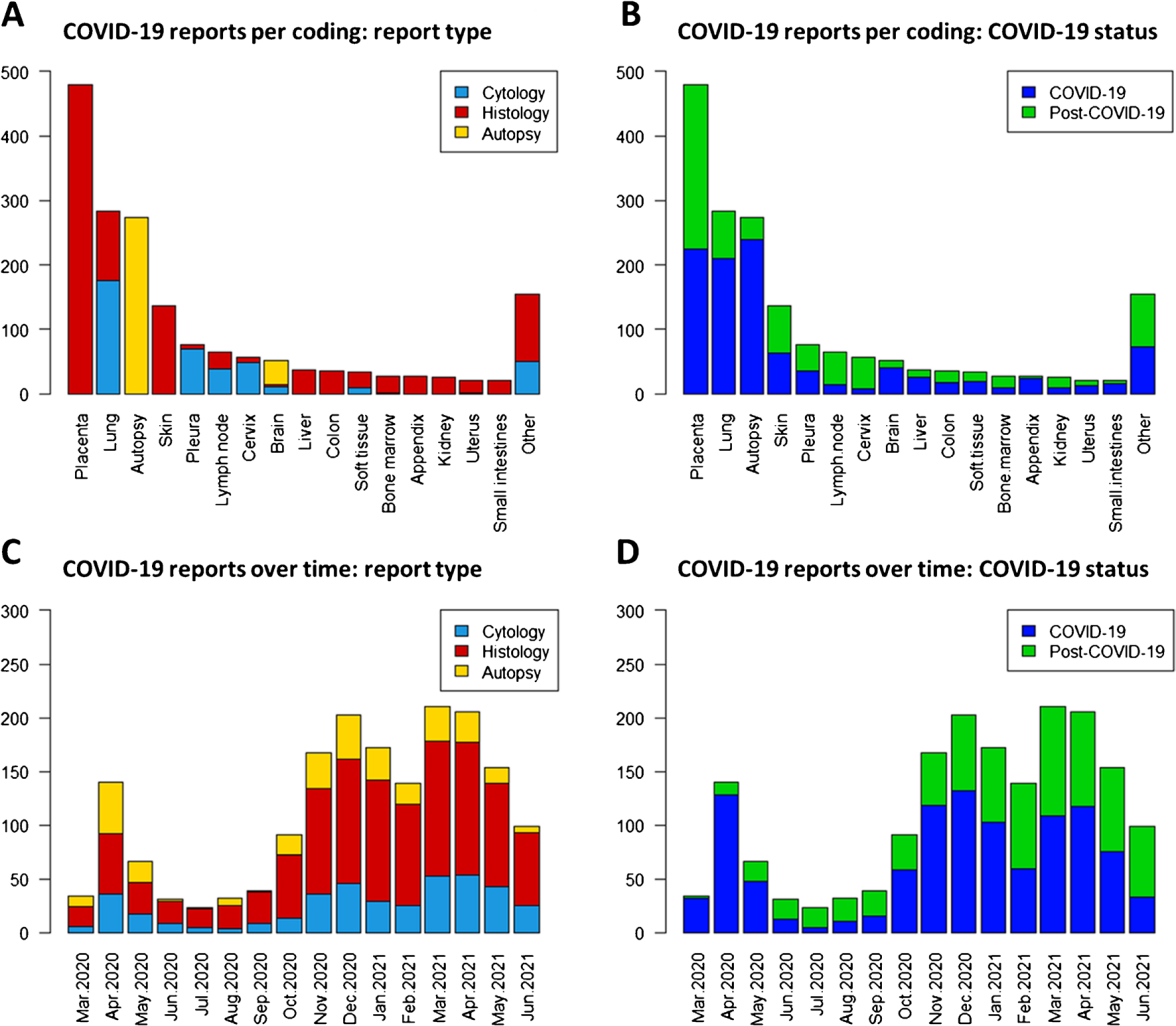
COVID-19-related pathology reports between January 2020 and June 2021 in the Netherlands. Top graphs show the total number of COVID-19-related reports, subcategorized by report type based on Palga coding (**A**, **C**) and COVID-19 status (**B**, **D**). Included in the category “Other” are as follows: adrenal gland, ascites, bone, breast, duodenum, esophagus, gallbladder, larynx, nasopharynx, ovary, pancreas, parathyroid gland, parotid gland, prostate, spleen, stomach, synovial fluid, testis, thyroid, trachea, ureter, urethra, urine bladder, vagina, and vascular. COVID-19-related reports over time (**C**, **D**) show three peaks corresponding with the first four pandemic waves in the Netherlands

A total of 311 eligible autopsy reports were extracted between January 2020 and June 2021, consisting of 269 full-body or post-mortal biopsy autopsies (lung, liver, and/or kidney), 37 brain autopsies, and 5 fetal autopsies of which the mother had COVID-19 during pregnancy. During this time period, the Netherlands faced three pandemic waves caused by the original Wuhan variant of SARS-CoV-2 in 2020 and a fourth wave of the alpha variant in 2021. The first wave occurred in the Netherlands between March and June of 2020. The second wave started in the autumn of 2020 and peaked on 31st October 2020, followed shortly by a third wave in December 2020. The fourth wave peaked in April 2021 [13]. These four pandemic waves are reflected by the autopsy rates per month during this period—albeit with a joint peak of the second and third wave—as their peaks correspond with the aforementioned pandemic waves (Fig. 2).

**Fig. 3:**
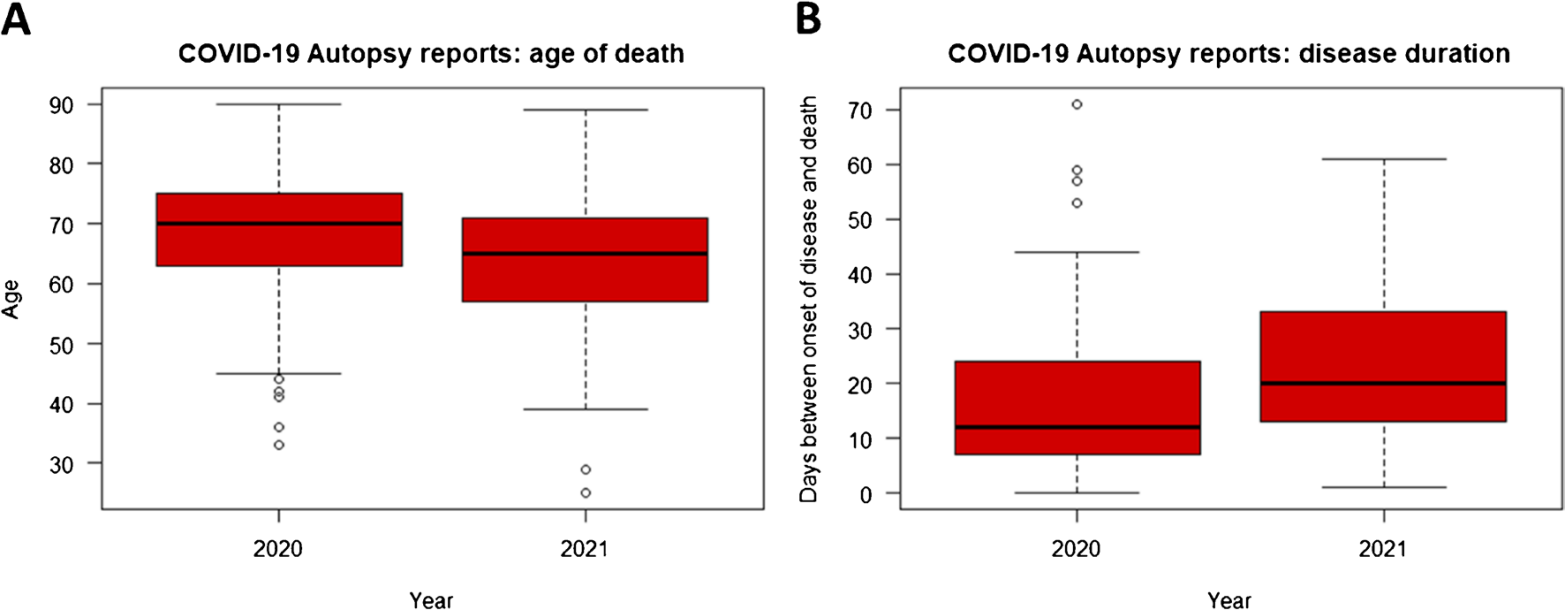
Difference in SARS-CoV-2 variants. A total of 269 eligible autopsy reports were included during 4 pandemic waves. The first three waves occurred in 2020 with prevalence of the Wuhan variant; the fourth wave occurred in 2021 with prevalence of the Alpha variant. **A** Age of death during 2020 was significantly higher (*p* < 0.001) compared to 2021 within the COVID-19-positive autopsies (*n* = 239). When post-COVID autopsies were also taken into account (*n* = 269), age of death remained significantly higher in 2020 (*p* = 0.002). **B** Disease duration was deducible from 139 autopsy reports, of which disease duration was significantly shorter from autopsies in 2020 compared to 2021. Significance of differences in age of death and disease duration were both determined by Wilcoxon signed-rank test

**Fig. 4:**
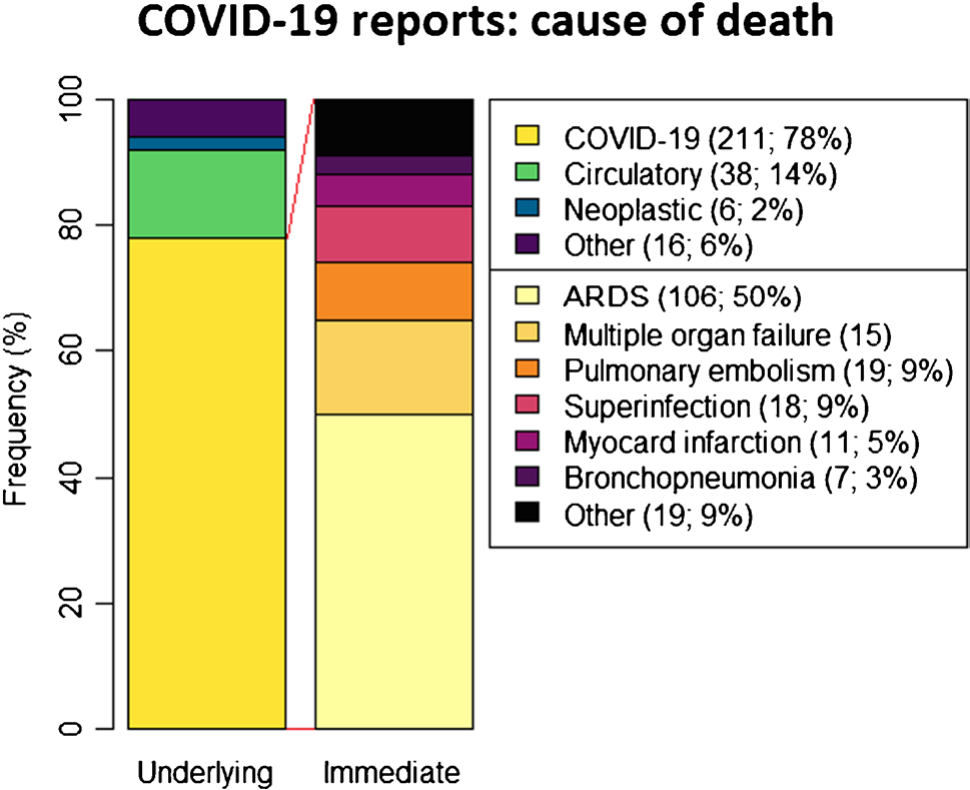
Cause of death in COVID-19 autopsy reports. Left column represents underlying cause of death of all included autopsy reports; right column represents immediate cause of death of autopsies with COVID-19 as underlying cause of death. Underlying cause of death was reported to be COVID-19 in 78% of all registered COVID-19 autopsies. The subgroup “Other” in underlying cause of death represents the following: digestive (*n* = 4), respiratory other than COVID-19 (*n* = 4), metabolic (*n* = 2), traumatic (*n* = 2), nervous (*n* = 1), and unknown (*n* = 1). The subgroup “Other” in the immediate cause of death bar depicts the following causes of death: hemodynamic shock (*n* = 5), ischemic heart disease (*n* = 5), cerebrovascular (*n* = 4), digestive (*n* = 2), arrhythmia (*n* = 1), nervous (*n* = 1), and asphyxia (*n* = 1)

The overall median age of death was 66 years (IQR 60–74), excluding 5 fetal autopsies. Interestingly, the mean age of death during the first three waves of the Wuhan variant was significantly higher than the mean age of autopsied patients during the 4th wave of the alpha variant (Fig. 3). This difference was significant for strictly COVID-19-positive autopsies (*n* = 239; *p* = 0.002) as well as COVID-19 positive autopsies in combination with post-COVID-19 autopsies (*n* = 269; *p* < 0.001). The male-to-female ratio was 2.15:1. No significant difference in age of death was found between males and females (male IQR, 61–74; female IQR, 60–74). The underlying cause of death was COVID-19 in 211 reports (78%), of which 106 (50%) had an immediate cause of death due to ARDS (acute respiratory distress syndrome). Other underlying causes of death and immediate causes of death in the COVID-19 subgroup are displayed in Fig. 4.

A total of 139 autopsy reports (52%) were eligible to determine the disease duration of COVID-19, of which 121 (86%) had COVID-19 at the time of death with a median disease duration of 16 days (IQR, 8–26 days). Reports from autopsies during the first three pandemic waves of the Wuhan variant in 2020 disclose significantly higher disease duration compared to autopsies during the alpha variant in 2021. This applies to COVID-19 patients taken together with post-COVID-19 patients (*p* = 0.012), as well as COVID-19 patients only (*p* = 0.049). There was no significant difference in disease duration between males and females (*p* = 0.92).

### COVID-19 pneumonia histology

**Fig. 5:**
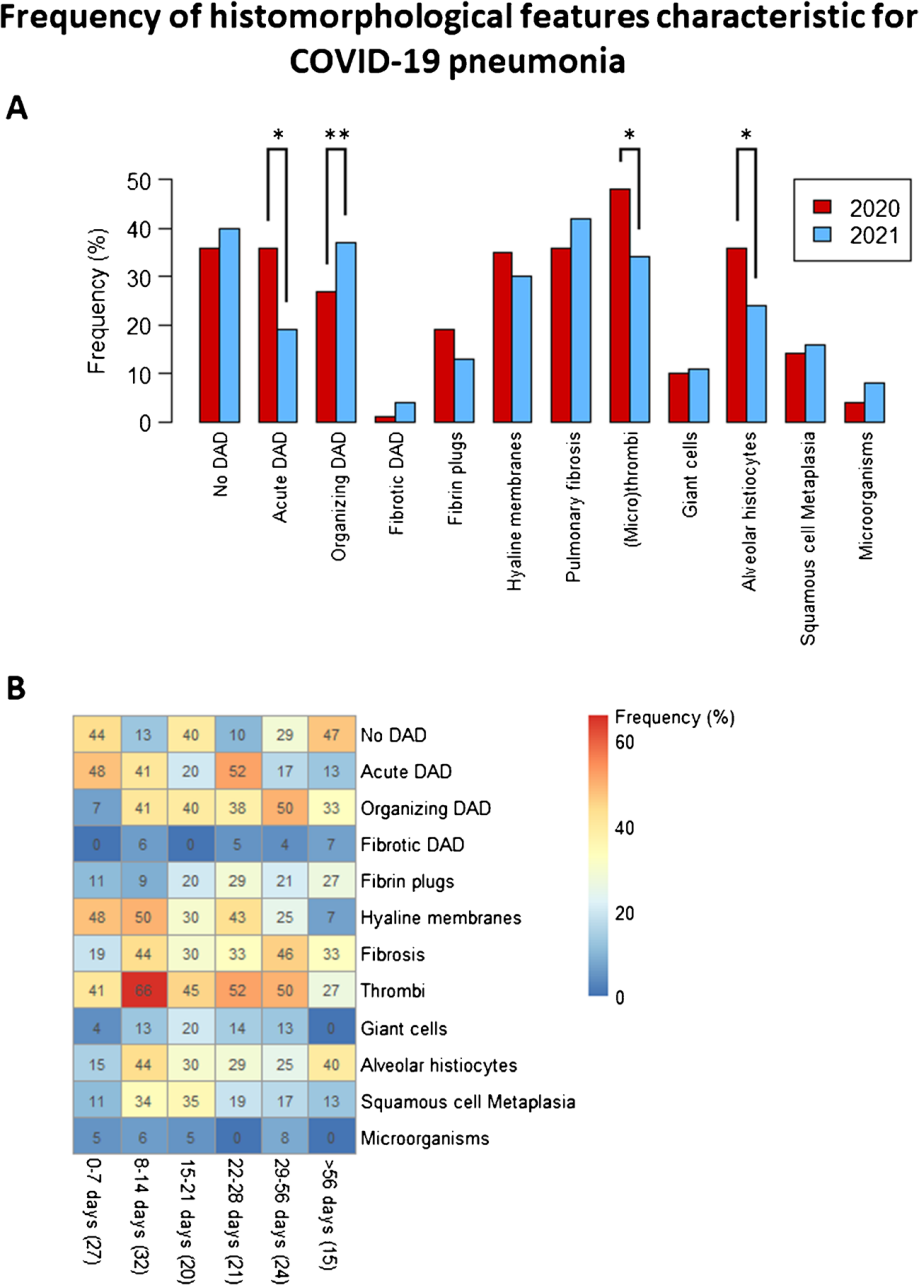
Frequency of histomorphological features characteristic for COVID-19 pneumonia stated in autopsy reports, displayed per year (**A**) and per duration of sickness (**B**). Features were considered absent if their presence was either denied or not mentioned at all. **A** In total, 362 pathology reports describing pulmonary histology were included from autopsy, biopsy, and excision reports; 211 reports originated from 2020 and 151 reports from 2021. The presence of acute DAD, (micro)thrombi, and alveolar histiocytes was significantly increased in pathology reports from 2020 compared to 2021 (*respective *p*-values < 0.001, 0.013, and 0.021). Organizing DAD was significantly more often mentioned in reports from 2021 (**p = 0.050). **B** Each cell displays the frequency (in %) of reports stating DAD phase or histopathological findings in reports. Each column depicts the amount of days between start of symptoms and death, with the total amount of reports in brackets. This figure depicts autopsy reports of which the duration of sickness was able to be deducted (139, 52% of all autopsy reports). Stratification by duration of sickness shows a slight increase in histologic features in the 2nd week of illness versus the 1st week

**Fig. 6:**
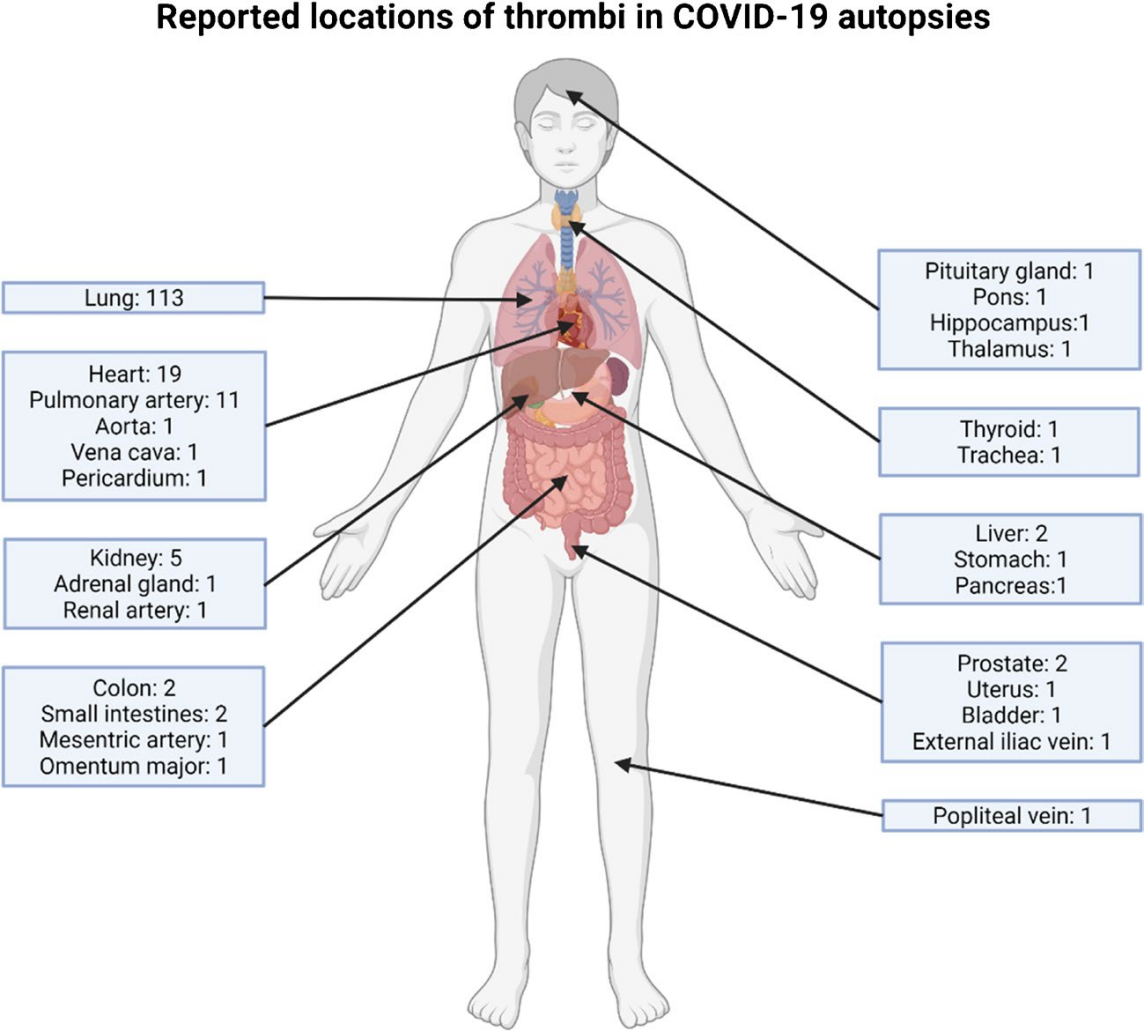
Reported locations of (micro)thrombi in COVID-19 autopsies. The presence of (micro)thrombi was reported in over half of the full-body autopsies (*n* = 137, 51%), of which the vast majority were pulmonary (micro)thrombi (82%). Intracranial thrombi were reported in 4 out of 37 COVID-19 brain autopsies

Descriptions of pulmonary histology were extracted from 362 pathology reports. These include 269 adult autopsies, 29 lung biopsies, and 24 excisions. Various histomorphological features characteristic for COVID-19 pneumonia have been reported in varying amounts such as DAD, pulmonary fibrosis, (micro)thrombi, and fibrin plugs, varying in presence depending on the report date (Fig. 5). The presence of acute DAD, (micro)thrombi, and alveolar histiocytes was significantly higher in reports from 2020 (*n* = 211) compared to reports from 2021 (*n* = 151). On the contrary, organizing DAD was significantly more often reported in reports from 2021. Regarding solely autopsies, the presence of (micro)thrombi was reported in over half of the full autopsies (*n* = 137; 51%), of which the majority were pulmonary (micro)thrombi (*n* = 113; 82%). Localization of reported thrombi in included autopsy reports is displayed in Fig. 6.

### Comorbidities in autopsies

Apart from (pulmonary) thrombosis, non-pulmonary comorbidities described in literature were found in autopsies as well. Abnormalities such as myocarditis, pericarditis, and cardiac amyloidosis have been reported in COVID-19 autopsies [14, 15] and were observed in our cohort as well, in varying amounts: myocarditis 19 (7%), pericarditis 4 (2%), cardiac amyloidosis 2 (1%). Acute tubular necrosis was observed in 26 (10%) of COVID-19 autopsies.

### Brain autopsies

Of the 47 brain autopsy reports that were retrieved, 37 (79%) stated the concerning patient had COVID-19 at the time of death, or COVID-19 was the cause of death. The male-to-female ratio within this group was 3.6:1. Over half of these reports (*n* = 21; 57%) describe a so-called “COVID-19 encephalitis,” meaning activation of microglia with microglial nodule formation and infiltration of T-lymphocytes without significant demyelination, necrosis, or axonal damage. The presence of intracranial (micro)thrombi was reported in 4 out of 37 reports, with thrombi in the pituitary gland, pons, hippocampus, and thalamus (Fig. 6). Cerebral amyloidosis was observed in 3 (6%) of brain autopsies, of which 1 case was known to have Alzheimer’s disease before death.

### Non-autopsy-related histopathology and cytology

COVID-19-related reports on non-autopsy histopathology and cytology totaled 1522, of which 782 (50%) reports stated the patient was COVID-19 positive at the time of specimen acquisition. The rate of non-autopsy COVID-19-related reports follows the pattern of pandemic waves as well as autopsies (Fig. 2). The vast majority of COVID-19-related material were placentas (*n* = 480; 32%), followed by lung (*n* = 286; 19%), skin (*n* = 137; 9%), and pleura (*n* = 81; 5%).

Non-autopsy lung histology (not cytology) were 96 reports, concerning of biopsies and lobectomies. Because a considerable amount of reports concerned standardized protocols with little to no description of pulmonary tissue, 55 (57%) of these reports were analyzed for histological features indicative for COVID-19 (Table 1). These reports were divided into two categories: COVID-19 positive and post-COVID-19. COVID-19-positive reports describe or frankly mention acute- and/or organizing pneumonia in 13 and 14 (36 and 39%) of reports, more often accompanied with features indicative for one or the other such as increased histiocytes (44%), pneumocyte hyperplasia (39%), fibrosis (33%), hyaline membranes (22%), and thrombi (17%). While post-COVID-19 lung tissue often mentions no diffuse alveolar damage (63%), fibrosis is reported in the majority of reports (53%), followed by increased macrophages (32%) and organizing pneumonia (26%).

**Table 1:** Frequency of diffuse alveolar damage (DAD) phase and histomorphological features characteristic for COVID-19 pneumonia stated in non-autopsy lung histology reports. Pathology reports depicting pulmonary lesions which did not reliably describe lung tissue were not included (e.g. standardized reports of lung carcinomas). Note that some pathology reports described multiple phases of DAD seen in one biopsy or resection

	COVID-19	Post-COVID-19
No DAD	6 (17%)	13
Acute DAD	14 (39%)	1
Organizing DAD	13 (36%)	5
Fibrotic DAD	4 (11%)	0
Firbrin plugs	6 (17%)	1
Hyaline membranes	8 (22%)	0
Fibrosis	12 (33%)	10
Thrombi	6 (17%)	1
Giant cell infiltrate	5 (14%)	0
Increased alveolar macrophages	16 (44%)	6
Squamous metaplasia	2 (6%)	0
Microorganisms	0 (0%)	1
Pneumocyte hyperplasia	14 (39%)	2
Total eligible reports	36	19

Of all COVID-19-related pathology reports regarding histology, placentas were the most reported with 224 COVID-19 positive and 256 post-COVID-19 cases. Various histopathological features described in COVID-19 placentas in literature were present in these as well [16, 17] (Table 2). These include fibrin deposits, acute and chronic inflammation, signs of ischemia, infarction, thrombosis, and intervillositis. The latter stood out by being reported relatively often in COVID-19-related reports: chronic histiocytic intervillositis was described in 21 out of all 480 (4.4%) histopathological reports concerning placentas from mothers with COVID-19. In a similar way, perniosis was reported in 8 out of 137 (5.8%) skin excisions or biopsies of COVID-19 patients. The presence of thrombi was reported in histopathology reports from biopsies and excisions of the following organs and structures (excluding autopsy reports): placenta, subdermal blood vessels, esophagus, stomach, small intestines, colon, and kidneys.

**Table 2:** Frequency of histopathological findings in COVID-19 placentas. Increased fibrin deposits were to most common histopathological feature in COVID-19 placentas as well as post-COVID-19 placentas

	COVID-19	Post-COVID-19
Signs of malperfusion	10 (4%)	15 (6%)
Increased fibrin depositions	43 (19%)	60 (23%)
Vasculopathy	13 (6%)	19 (7%)
Signs of ischemia	22 10%)	56 (22%)
Acute inflammation	37 (17%)	45 (18%)
Chronic inflammation	17 (8%)	15 (6%)
Chorangiosis	5 (2%)	4 (2%)
Thrombosis	11 (5%)	4 (2%)
Necrosis	7 (3%)	4 (2%)
Intervillositis	14 (6%)	7 (3%)
Intervillary hemorrhage	4 (2%)	14 (5%)
Infarction	29 (13%)	28 (11%)
Total	224	256

## Discussion

Pathology reports of Dutch COVID-19 autopsies, as well as clinical histopathology and cytology of (former) COVID-19 patients, were retrieved to form a collection of COVID-19-related pathology reports. This work shows that pathology reports during the first three waves of the Wuhan variant (2020) describe significantly more acute DAD, thrombi, and alveolar histiocytes compared to reports from the fourth wave of the alpha variant (2021) which in turn shows significantly more organizing DAD. Furthermore, this collection of reports also highlights thrombosis and other comorbidities of COVID-19 in other organs besides the lungs. Although this collection is limited by the restriction for pathology requisitions, it is able to build up data over time by adding new excepts of (former) COVID-19 patients to potentially serve as a post-viral surveillance tool for research on long-term effects of COVID as well as the severity of different variants of SARS-CoV-2.

### Findings from COVID-19 autopsy reports

The amount of COVID-19 autopsies included in this collection (*n* = 269, excluding brain and fetal autopsies) was a mere fraction of all COVID-19 deaths in the Netherlands. Between January 2020 and June 2021, a total of 17,748 COVID-19 deaths have been registered in the Netherlands, meaning autopsy was performed on 1.5% of all Dutch COVID-19 deaths [18]. COVID-19 adult autopsies took up roughly 6% of all adult autopsies in the Netherlands, which are estimated to be around 4400 during this time period based on retrieved autopsy data by Palga. Although autopsy was performed on a small percentage of COVID-19 deaths, the distribution of COVID-19 autopsy reports per month shows a substantial overlap with the first four waves of COVID-19 infections in the Netherlands. Despite the fact that included reports did not state the causative virus variant, it is reasonable to assume that autopsies, histopathology, or cytology obtained during specific pandemic waves are largely linked to prevalent variants of SARS-CoV-2. For instance, between March and June 2021, the alpha variant of SARS-CoV-2 had a prevalence between 83.9 and 97.2% [19] in the Netherlands. Autopsy reports from 2021 during the pandemic wave of the alpha variant state a significantly younger age of death and longer duration of disease compared to reports from 2020 during waves of the original Wuhan strain. Our findings are in line with data from the Central Bureau of Statistics in the Netherlands: 5.2% of COVID-19 deaths were patients of 65 years old or below in 2020 compared to 7.8% in 2021 [20]. A possible explanation for the difference in age could be that patients with variant alpha COVID-19 show a more severe course during ICU admission [21–23], possibly enhanced by suboptimal treatment of COVID-19. This is also reflected in histopathology reports concerning lung histology from the same year: histomorphological changes portraying acute damage (acute DAD and increase of alveolar histiocytes) as well as (micro)thrombi were significantly more present in reports from 2020 compared to reports from 2021. Contrariwise, organizing DAD was reported significantly more often in reports from 2021 compared to 2020, fitting with prolonged disease duration. However, a bias by intent to perform autopsy may have occurred. As COVID-19 pathology was more known in 2021, clinicians may have requested autopsy relatively more often in cases with misunderstood declining condition, including younger patients. Thus, autopsy reports may provide additional information on disease severity, although they should be interpreted with caution.

Other non-pulmonary findings which were described to be present in COVID-19 autopsies such as myocarditis, cardiac amyloidosis, and acute tubular necrosis were reported as well, but less often is described in literature [14, 15]. There are a few possible reasons for this. Firstly, no standardized form for (COVID-19) autopsies was in place, meaning pathologists with little to no experience with COVID-19 autopsies could miss these abnormalities. Secondly, the cause of death was not always related to COVID-19; this was especially more often the case for post-COVID autopsies. While we believe the total amount of available reports proves to be sufficient to provide a comprehensive overview of common findings in COVID-19, we also believe that data could be refined if reports provided more information on patient history and clinical state. This context would especially provide essential information regarding non-autopsy histology reports.

### Findings from COVID-19 histology reports

Lung tissue from non-autopsy reports also describes the presence of certain features of acute and organizing pneumonia, or frankly mentions its DAD state. Compared to COVID-19 autopsies, COVID-19 lung tissue shows overall less of these features. A simple explanation would be that COVID-19 autopsies often depict the most severe state of COVID-19 pneumonia. The difference in histology between COVID-19-positive and post-COVID-19 lung tissue is interesting, as fibrosis is relatively more often observed in the latter. Pulmonary fibrosis in post-COVID-19 patients is frequently reported and is accompanied by persistent symptoms of dyspnea, fatigue, myalgia, and considerable decline of pulmonary function [24, 25]. This collection of reports shows similar findings in a subgroup of COVID-19 survivors which may indicate an increase in pulmonary fibrosis in the years to come.

Besides pulmonary thrombi, this collection of reports points out thrombosis in other organs and structures. Autopsy reports state the presence of thrombi mostly not only in lung tissue, but also in other organs such as the heart, kidneys, and intestines. Interestingly, the presence of (micro)thrombi in these organs was also described in histopathology reports from resections and biopsies during the clinical phase. This further underscores the systemic pro-coagulant effect of COVID-19. Other comorbidities seemingly related to COVID-19 were highlighted as well. For example, chronic histiocytic intervillositis was present in 4.4% of all included COVID-19 placentas. This illness was reported to affect 6 out of 10,000 analyzed placentas beyond 12 weeks gestation [26], a far lower rate compared to our findings. Other findings reported in COVID-19 placentas such as signs of ischemia, fibrin deposits, infarction, and thrombosis were reported in our query result as well [16, 17]. However, our cohort reports these features to a lesser degree which may be related to the severity of COVID-19 of selected cases. Perniosis, a rare inflammatory condition of the skin, was also stated in 5.8% of histopathology reports regarding skin biopsies and excisions of COVID-19 patients. These ratios may even be underestimations as 42% (*n* = 235) and 38% (*n* = 281) from respectively placenta and skin pathology reports did not show the inquiry. This loss on information of COVID-19 status may have led to false negative pathology reports. Still, both comorbidities have been described to occur as manifestations of SARS-CoV-2 infection [27–30]. The inclusion of pathology reports on patient material in the clinical phase of COVID-19 in this collection adds to understanding the manifestation of COVID-19 in different stages of disease and severity.

### Advantages of this COVID-19 pathology report collection

Firstly, data from pathology reports contain substantially more detailed information compared to other surveillance tools, especially concerning COVID-19 deaths. For example, the Dutch Central Bureau of Statistics (CBS) keeps track of COVID-19 deaths, defined by COVID-19 being the underlying cause of death. However, this information is restrictive as details on complications, comorbidities, immediate cause of death, and other clinical information are still not taken into account. Furthermore, disclosed COVID-19 deaths are only included if the deceased patient tested positive for SARS-CoV-2, missing a substantial number of untested COVID-19 deaths [31]. Although this collection provides information on a portion of COVID-19 deaths, it does so in a more comprehensive and inclusive manner.

Secondly, Palga allows for the inclusion of a nationwide array of reports. All laboratories in the Netherlands already use an automated registry of pathology reports without subsidiary work of pathologists and clinicians. In Germany, a web-based registry was established to gather information on COVID-19 histology from autopsy reports [32]. While it had advantages compared to our collection (which will be discussed in the *limitations* section), a drawback was that this particular registry was not automated. As the Palga database updates automatically every week, retrieval of data takes not more than a week, thus COVID-19 pathology reports may gradually accumulate. Palga also allows for the inclusion of cytology and histopathology reports besides autopsies, securing data on COVID-19 during the clinical phase. These histopathology reports were helpful in providing data on pulmonary damage in less severe stages of COVID-19. Furthermore, it allowed less obtrusive comorbidities in other organs beside the lungs to emerge, providing insight in the pathophysiology of COVID-19. The previously mentioned web-based registry was able to gather information on COVID-19 histology but was limited to only samples related to autopsy cases [32]. To our knowledge, this is the first full nationwide registration of COVID-19-related pathology reports, including autopsies, histopathology, and cytology.

Thirdly, follow-up reports of former COVID-19 patients were identified by Palga, even if reports did not mention COVID-19 status or were not coded accordingly. This follow-up feature proved to be useful for identifying a substantial number of post-COVID-19 reports, as 90% of all identified follow-up reports did not mention the patient was a former COVID-19 patient. For reports only concerning lung tissue or cytology, this percentage was 71%. Successive reports of (former) COVID-19 patients can be utilized to gather information on long-term effects of COVID-19.

Fourthly, The Palga database is linked to many other databases and registries such as the National Cancer Registry (NKR), Statistics Netherlands (CBS), Pharmo, StOET, SHM, PLCRC, LifeLines, and AOCR [33].

Lastly, this collection is able to expand, as the processed data without false positive reports allows for future pathology reports from (former) COVID-19 positive to be identified by Palga. Acquired reports can be sorted by different epidemiological parameters to highlight the effects of (long) COVID-19 in certain populations (e.g., age, sex, region, data of specimen retrieval, specific medical history). Over time, valuable data regarding long-term effects of COVID-19 and potential upcoming SARS-CoV-2 variants may emerge.

### Limitations

The foremost limitation was that a pre-existing automated registry was used which was not specifically designed for COVID-19-related reports. This meant that COVID-19 status, along with clinical information, was not directly imported by clinicians and had to be established based on either inquiry, pathology report, or coding. Clinicians would most likely report COVID-19 status if they deemed this information to be important for pathologists. Consequently, it is fair to assume that a proportion of COVID-19 reports are missing from this collection. This gap in information may be reduced in the future when long COVID becomes more apparent. In addition, 40% of the labs did not disclose information stated in the wording of the initial request, leading to a potential loss of information; macroscopy, microscopy, and conclusion were disclosed however. We rely on the information that is available in the Palga database, but more labor intense work could still gather this information when needed. Still, it is feasible that pathologists would address COVID-19 status in the pathology reports if relevant to the diagnosis or even when doubting its relevance. As the content of all included pathology reports was available, excluded pathology reports without available inquiry would be less likely to contribute to identifying comorbidities of COVID-19.

As stated before, another limitation was that autopsy reports were not standardized, which may have led to underreporting of certain histopathological features. Previous registries or records of findings in COVID-19 autopsies had standardized forms for reporting such findings [32, 34]. This way, pathologists would be reminded to search for certain features. Additionally, specific information regarding viral load per organ and virus variant were not reported in most cases and could not consistently be relied on.

Reports had to be manually screened for COVID-19 status to exclude false positive reports, which proved to be a time-consuming process. This process was essentially bypassed in another registry, which required to have pathologists fill in a form asking for specific findings which was automatically translated into utilizable data [32]. Our initial approach was automatic selection based on coding attached to reports. However, the majority of reports concerning COVID-19-positive patients were not coded as such by pathologists. Less than half of registered COVID-19 autopsies (43%) were specifically coded to include COVID-19. This percentage was lower for non-autopsy reports of COVID-19-positive material (11% of histopathology reports and 12% of cytology reports). This may be caused by COVID-19 having no (direct) relationship to the diagnosis. For future inclusion of reports, structured reporting or perhaps the use of artificial intelligence may speed up the selection process.

## Conclusion

In summary, this nationwide collection of autopsy, histology, and cytology reports provides detailed information regarding the effects of COVID-19 not only in lung tissue but also in other organs. It also demonstrates that some of these effects are also reported in post-COVID-19 patients which may be related to long COVID. Data from this collection may support medical research and development of guidelines on potential new variants of SARS-CoV-2. As consecutive pathological reports of these patients can easily be identified and retrieved, this collection of reports could function as the basis for future studies regarding long-term effects of COVID-19.

## Data Availability

The data that support the findings of this study are available from the corresponding author upon reasonable request.

## References

[1] Organisation WH. WHO coronavirus (COVID-19) dashboard 2022 [Available from: https://covid19.who.int/.

[2] Polak SB, Van Gool IC, Cohen D, von der Thüsen JH, van Paassen J (2020). A systematic review of pathological findings in COVID-19: a pathophysiological timeline and possible mechanisms of disease progression. Mod Pathol.

[3] Sharma P, Ng JH, Bijol V, Jhaveri KD, Wanchoo R (2021). Pathology of COVID-19-associated acute kidney injury. Clin Kidney J.

[4] Batah SS, Fabro AT (2021). Pulmonary pathology of ARDS in COVID-19: a pathological review for clinicians. Respir Med.

[5] Hariri LP, North CM, Shih AR, Israel RA, Maley JH, Villalba JA (2021). Lung histopathology in coronavirus disease 2019 as compared with severe acute respiratory syndrome and H1N1 influenza: a systematic review. Chest.

[6] Sudre CH, Murray B, Varsavsky T, Graham MS, Penfold RS, Bowyer RC (2021). Attributes and predictors of long COVID. Nat Med.

[7] Taquet M, Dercon Q, Luciano S, Geddes JR, Husain M, Harrison PJ (2021). Incidence, co-occurrence, and evolution of long-COVID features: a 6-month retrospective cohort study of 273,618 survivors of COVID-19. PLoS Med.

[8] Arnold DT, Hamilton FW, Milne A, Morley AJ, Viner J, Attwood M (2021). Patient outcomes after hospitalisation with COVID-19 and implications for follow-up: results from a prospective UK cohort. Thorax.

[9] Cha MH, Regueiro M, Sandhu DS (2020). Gastrointestinal and hepatic manifestations of COVID-19: a comprehensive review. World J Gastroenterol.

[10] Kaya G, Kaya A, Saurat JH (2020). Clinical and histopathological features and potential pathological mechanisms of skin lesions in COVID-19: review of the literature. Dermatopathology (Basel).

[11] Ng JH, Bijol V, Sparks MA, Sise ME, Izzedine H, Jhaveri KD (2020). Pathophysiology and pathology of acute kidney injury in patients with COVID-19. Adv Chronic Kidney Dis.

[12] Casparie M, Tiebosch AT, Burger G, Blauwgeers H, van de Pol A, van Krieken JH (2007). Pathology databanking and biobanking in The Netherlands, a central role for PALGA, the nationwide histopathology and cytopathology data network and archive. Cell Oncol.

[13] Varianten van het coronavirus SARS-CoV-2: Rijksintituut voor Volksgezondheid en Milieu; 2022 [Available from: rivm.nl/coronavirus-covid-19/virus/varianten

[14] Almamlouk R, Kashour T, Obeidat S, Bois MC, Maleszewski JJ, Omrani OA (2022). COVID-19-Associated cardiac pathology at the postmortem evaluation: a collaborative systematic review. Clin Microbiol Infect.

[15] Menezes RG, Rizwan T, Saad Ali S, Hassan W, Khetpal A, Aqil M (2022). Postmortem findings in COVID-19 fatalities: a systematic review of current evidence. Leg Med (Tokyo).

[16] Joshi B, Chandi A, Srinivasan R, Saini SS, Prasad GRV, Puri GD (2022). The placental pathology in coronavirus disease 2019 infected mothers and its impact on pregnancy outcome. Placenta.

[17] Garg R, Agarwal R, Yadav D, Singh S, Kumar H, Bhardwaj R (2023). Histopathological changes in placenta of severe acute respiratory syndrome coronavirus 2 (SARS-Cov-2) infection and maternal and perinatal outcome in COVID-19. J Obstet Gynaecol India.

[18] Coronavirus Casus Netherlands: Dadax Limited; 2022 [Available from: worldometers.info/coronavirus/country/netherlands/

[19] Rijksoverheid. Varianten van het coronavirus 2023 [Available from: https://coronadashboard.rijksoverheid.nl/landelijk/varianten

[20] Netherlands S. Sterfte aan COVID-19 per maand en leeftijdsgroep 2023 [updated 8–2–2023. Available from: https://www.cbs.nl/nl-nl/maatwerk/2023/06/sterfte-aan-covid-19-per-maand-en-leeftijdsgroep

[21] Florensa D, Mateo J, Spaimoc R, Miret C, Godoy S, Solsona F (2022). Severity of COVID-19 cases in the months of predominance of the Alpha and Delta variants. Sci Rep.

[22] Vassallo M, Manni S, Klotz C, Fabre R, Pini P, Blanchouin E et al (2021) Patients admitted for variant alpha COVID-19 have poorer outcomes than those infected with the old strain. J Clin Med 10(16) 10.3390/jcm10163550 PMC8396910 34441844

[23] Donati S, Corsi E, Maraschini A, Salvatore MA (2022) It OSSC-WG. SARS-CoV-2 infection among hospitalised pregnant women and impact of different viral strains on COVID-19 severity in Italy: a national prospective population-based cohort study. Bjog 129(2):221–31 10.1111/1471-0528.16980 PMC8652503 34687585

[24] Hama Amin BJ, Kakamad FH, Ahmed GS, Ahmed SF, Abdulla BA, Mohammed SH (2022). Post COVID-19 pulmonary fibrosis; a meta-analysis study. Ann Med Surg (Lond).

[25] Hirawat R, Jain N, Aslam Saifi M, Rachamalla M, Godugu C (2023). Lung fibrosis: Post-COVID-19 complications and evidences. Int Immunopharmacol.

[26] Contro E, deSouza R, Bhide A (2010). Chronic intervillositis of the placenta: a systematic review. Placenta.

[27] Mao Q, Chu S, Shapiro S, Young L, Russo M, De Paepe ME (2022). Placental SARS-CoV-2 distribution correlates with level of tissue oxygenation in COVID-19-associated necrotizing histiocytic intervillositis/perivillous fibrin deposition. Placenta.

[28] Schwartz DA, Baldewijns M, Benachi A, Bugatti M, Collins RRJ, De Luca D (2021). Chronic histiocytic intervillositis with trophoblast necrosis is a risk factor associated with placental infection from coronavirus disease 2019 (COVID-19) and intrauterine maternal-fetal severe acute respiratory syndrome coronavirus 2 (SARS-CoV-2) transmission in live-born and stillborn infants. Arch Pathol Lab Med.

[29] Cappel MA, Cappel JA, Wetter DA (2021). Pernio (Chilblains), SARS-CoV-2, and COVID toes unified through cutaneous and systemic mechanisms. Mayo Clin Proc.

[30] McCleskey PE, Zimmerman B, Lieberman A, Liu L, Chen C, Gorouhi F (2021). Epidemiologic analysis of chilblains cohorts before and during the COVID-19 pandemic. JAMA Dermatol.

[31] What’s meant by number of reported deaths? : Rijksoverheid; 2021 [Available from: https://coronadashboard.government.nl/artikelen/wat-zegt-het-aantal-meldingen-sterfgevallen

[32] von Stillfried S, Bülow RD, Röhrig R, Boor P (2022) German Registry of Covid-19 Autopsies DC. First report from the German COVID-19 autopsy registry. Lancet Reg Health Eur 15:100330 10.1016/j.lanepe.2022.100330 PMC9073019 35531493

[33] Palga. Palga [Available from: https://www.palga.nl/

[34] Schwab C, Merle U, Schirmacher P, Longerich T (2023). Lethality of SARS-CoV-2 infection-a comparative autopsy study focusing on COVID-19 development and virus variants. Histopathology.

